# Fungal secondary metabolites rasfonin induces autophagy, apoptosis and necroptosis in renal cancer cell line

**DOI:** 10.1080/21501203.2016.1181114

**Published:** 2016-05-09

**Authors:** Hui Sun, Weijun Wang, Yongsheng Che, Xuejun Jiang

**Affiliations:** a State Key Laboratory of Mycology, Institute of Microbiology, Chinese Academy of Sciences, Beijing, China; b University of Chinese Academy of Sciences, Beijing, China; c Beijing Institute of Pharmacology & Toxicology, Beijing, China

**Keywords:** Rasfonin, autophagy, apoptosis, necroptosis, RIP1

## Abstract

Rasfonin (A304) is a fungal natural product isolated from the fermentation substrate of *Talaromyces* sp. 3656-A1, which was named according to its activity against the small G-protein Ras. In a former study, we demonstrated that it induced autophagy and apoptosis; however, whether rasfonin activated necroptosis remained unknown. Moreover, the interplay among different cell death processes induced by rasfonin was unexplored. In the present study, we revealed that, in addition of promoting autophagy and caspase-dependent apoptosis, rasfonin also activated necroptosis. Nectrostatin-1 (Nec-1), an inhibitor of necroptosis, affected rasfonin-induced autophagy in a time-dependent manner concurring with an increased caspase-dependent apoptosis. The aforementioned results were confirmed by knockdown of receptor-interacting protein 1 (RIP1), a crucial necrostatin-1-targeted adaptor kinase mediating cell death and survival. Taken together, the data presented indicate that rasfonin activates various cell death pathways, and RIP1 plays a critical role in rasfonin-induced autophagy and apoptosis.

## Introduction

Rasfonin (A304) is a natural product isolated from the fermented mycelium of *Talaromyces* sp. 3656-A1, a derivative of a class of fungal secondary metabolites known as 2-pyrones (Tomikawa et al. 2000). 2-pyrones have been previously reported to have a broad range of biological activities (Hilgeroth 2002; Marrison et al. 2002; McGlacken and Fairlamb 2005). Recently, rasfonin was shown to induce the death of ras-mutated pancreatic tumour (Panc-1) cells (Xiao et al. 2014). In our former study, we have demonstrated that rasfonin induced both apoptosis and macroautophagy (hereafter called autophagy), and the inhibition of autophagy either chemically or genetically reduced rasfonin-dependent apoptosis (Lu et al. 2015).

Based on distinct cell morphology, programmed cell death (PCD) is divided into three major types: apoptosis, autophagic cell death and programmed necrosis (necroptosis) (Levine and Yuan 2005; Maiuri et al. 2007; Kroemer and Levine 2008). Accumulated evidence suggests the existence of several molecular connections among them (Edinger and Thompson 2004). In response to specific perturbations, the same input signal can cause cells to change from one cell death manifestation to another, with a mixed type of cell death observed in some cases.

Autophagy is an evolutionarily conserved intracellular membrane trafficking process that is involved in the delivery of cytoplasmic contents and organelles to the lysosomes for degradation (Yang and Klionsky 2010). LC3 (a mammalian homolog of yeast Atg8) is the most widely monitored autophagy-related protein. LC3-II is the only protein marker that is reliably associated with completed autophagosomes (Klionsky et al. 2016). Although it initially serves as a cell survival mechanism, autophagy, if overactivated and allowed to go to excess, will eventually lead to cell death by depletion of the cell’s organelles and critical proteins (Levine and Yuan 2005).

Necroptosis is also named programmed necrosis. It has its unique signalling pathway, which requires the involvement of receptor interaction protein kinase 1 and 3 (RIP1 and RIP3), and can be specifically inhibited by necrostatins (Holler et al. 2000; Galluzzi et al. 2009). Necrostatin-1 (Nec-1) was initially defined as an inhibitor of necroptosis because of the specific inhibitory effect of RIP1. Further studies have revealed the underlying mechanism with greater detail: the inactivation of RIP1 blocks the recruitment and interaction of RIP3 and RIP1, thus inhibiting the formation of necrosome and necroptosis (Degterev et al. 2008).

In the present study, we found that, in addition to inducing autophagy and apoptosis, rasfonin also activated programmed necrosis, and RIP1 was involved in rasfonin-induced autophagy and caspase-dependent apoptosis.

## Results

### Nec-1 decreases rasfonin-induced cell viability loss, apoptosis and necrosis

**Figure 1.**
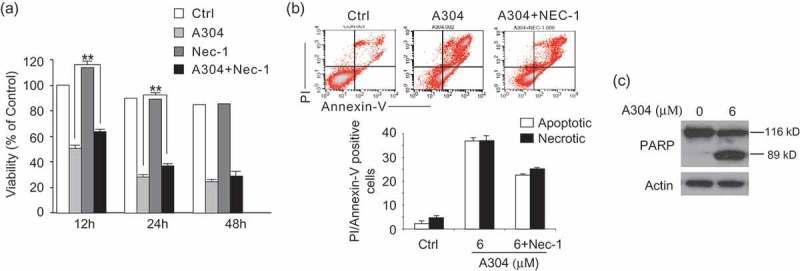
Nec-1 decreases rasfonin-induced cell viability loss attenuates rasfonin-induced PARP-1 cleavage. (a) ACHN cells were treated with rasfonin (6 μM) in the presence of Nec-1 (30 μM) for up to 48 h; cell viability was analysed by MTS assay as described in Materials and Methods. Data are presented as mean ± SD and are representatives of three independent experiments. Each performed in triplicate, and the data was analysed by *t*-test. Single asterisk denotes that the group is statistically different from the control groups (*p* < 0.05), whereas double asterisk means *p* < 0.01. (b) Following treatment of the cells with rasfonin (6 μM) and Nec-1 (30 μM) for 12 h, the apoptosis and necrosis induced were determined by flow cytometry. Apoptotic: AV-positive and PI-negative; necrotic: PI-positive. The data are presented as mean ± SD from three independent experiments. (c) The cells were treated with rasfonin (6 μM) up to 12 h, cell lysates were prepared and analysed by immunoblotting using the indicated antibodies. Actin was used as loading control.

In our previous study, we revealed that rasfonin induces both autophagy and apoptosis, and cell viability was decreased in both tumor cells (Figure 1(a), Supplemental data) and relatively normal cell lines (Supplemental data), but whether it activates necroptosis was unknown. Nec-1, an inhibitor of necroptosis, was found to decrease rasfonin-induced cell viability loss in ACHN cells (Figure 1(a)). In flow cytometry assay, we observed that rasfonin was able to activate apoptosis and necrosis, both of which were inhibited in the presence of Nec-1 (Figure 1(b)). Immunoblotting analysis revealed that rasfonin induced cleavage of PARP-1 (Figure 1(c)). PARP-1 is one of the main cleavage targets of caspase-3 *in vivo*, and its cleavage serves as a marker of cells undergoing apoptosis (Amé et al. 2004; Andrabi et al. 2006), suggesting the activation of caspase-dependent apoptotic pathway.

### Rasfonin can activate all three types of programmed cell death

**Figure 2.**
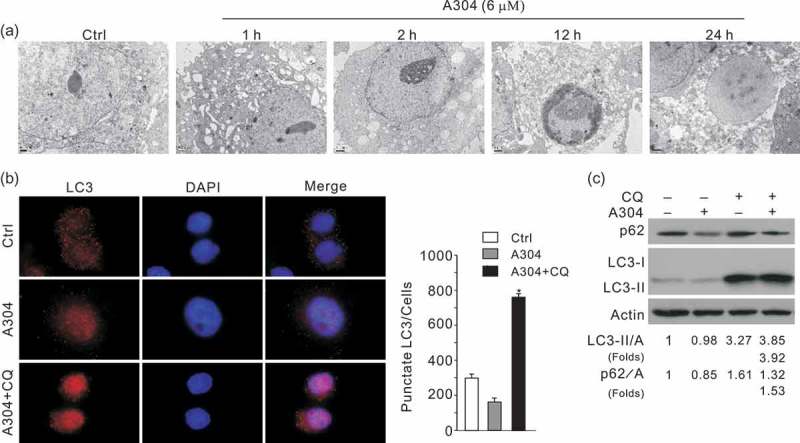
Rasfonin induce autophagy in ACHN cells. (a) Electron microscopy was applied in ACHN cells following treatment of rasfonin (6 μM) up to 24 h. (b) Immunofluorescence using LC3 antibody was performed on ACHN cells following the treatment of rasfonin (6 μM) in the presence or absence of CQ (15 μM) for 2 h. The number of the punctate of LC3 in each cell was counted, and at least 50 cells were included for each group. Data representing the mean ± SD is shown in graph. (c) ACHN cells were treated with rasfonin (6 μM) for 2 h in the presence or absence of CQ (15 μM). The cells were lysed and subjected to immunoblotting with the antibodies indicated.

Electron microscopy (EM), one of the most convincing approaches to detect autophagy (Klionsky et al. 2012), was used to determine whether rasfonin enhances autophagy. Compared with the control, an obvious accumulation of membrane vacuoles was observed in rasfonin-treated ACHN cells at the 1 h time point (Figure 2(a)); however, autophagosomes appeared not to be further increased at the 2 h time point (Figure 2(a)). Upon longer time of stimulation, rasfonin was found to induce the morphology of either apoptosis or necrosis (Figure 2(a)). Aforementioned results indicated that rasfonin was able to induce autophagy, apoptosis and necrosis. Interestingly, we observed rasfonin reduced punctate staining of LC3. Chloroquine (CQ), which blocks the fusion between autophagosome and lysosome and is often used in detection of autopohagic flux (Kovacs and Seglen 1982; Grumati et al. 2010) combined with rasfonin increases the punctate staining (Figure 2(b)), suggesting that rasfonin enhanced autophagic flux. Immunostaining results were confirmed by immunoblotting assay (Figure 2(c)). Thus, all three methods revealed that rasfonin indeed activated autophagic process and could be used as an autophagy inducer in future study.

### Nec-1 affected rasfonin-induced autophagy in a time-dependent manner and promoted the induced caspase-dependent apoptosis

**Figure 3.**
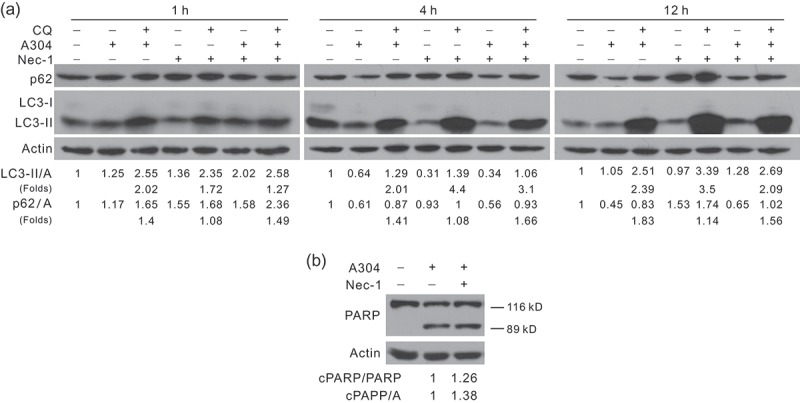
Nectrostatin-1 (Nec-1) attenuates rasfonin-induced autophagy with the cleaved PARP-1 (cPARP) decreased. After treatment of rasfonin with Nec-1 in the presence of CQ for 1, 4 and 12 h (a), ACHN cell lysates were analysed by immunoblotting with the antibodies indicated. Relative levels of LC3-II were calculated and presented below the blots. Actin was used as loading control. (b) The cleavage of PARP-1 was detected at 12 h by Western blotting.

RIP1 has been demonstrated to involve in regulation of C42-induced autophagy, thus, we next examined whether this adaptor regulated autophagy induced by rasfonin. Nec-1, an inhibitor of RIP1, appeared to affect rasfonin-activated autophagy in a time-dependent manner. While Nec-1 attenuated rasfonin-induced autophagy at both 1 and 12 h time points, it increased autophagic flux at the 4 h time point evaluating LC3-II accumulation and p62 degradation in the presence of CQ (Figure 3(a)). SQSTM1/p62 is a selective substrate of autophagy and is often utilised as a marker to monitor autophagy (Klionsky et al. 2012). During autophagic process, p62 was degraded and CQ usually blocked its degradation. Moreover, we observed that Nec-1 alone was able to promote autophagy (Figure 3(a)). In addition, Nec-1 was found to increase rasfonin-induced PARP-1 cleavage (Figure 3(b)), suggesting that the inhibition of necroptosis switched cell death to caspase-dependent apoptosis.

### RIP1 deprivation decreases rasfonin-induced autophagic flux and increases PARP-1 cleavage

**Figure 4.**
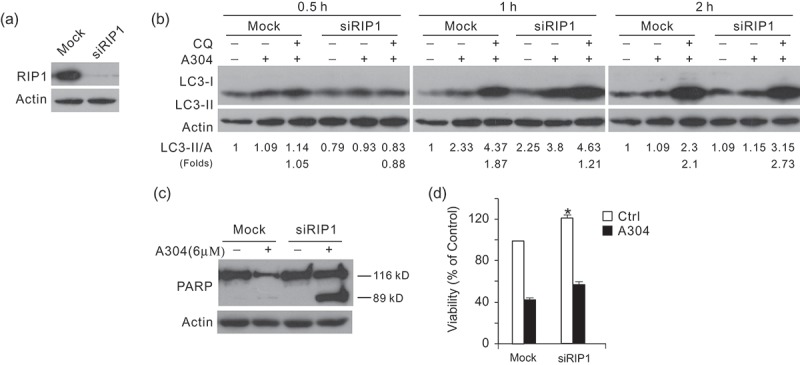
Knockdown of receptor-interacting protein 1 (RIP1) decreases rasfonin-dependent autophagy, but stimulate the rasfonin-induced cleavage of PARP-1. (a) ACHN cells were transfected with the RIP1 siRNA for 48 h. After following treatment of rasfonin (6 μM) in the presence or absence of CQ (15 μM) up to 2 h, the lysates were analysed by immunoblotting. Relative levels of LC3-II were calculated and presented below the blots. Similar experiments repeated three times. (b) Up to 12 h, cPARP was detected. (c) MTS assay were performed to detect the viability of ACHN cells for 24 h.

To confirm results obtained with Nec-1, we knocked down RIP1 and investigated the induced autophagy upon stimulation of rasfonin. Utilising immunoblotting, we found that RIP1 was sufficiently silenced, by 90% reduction in its expression (Figure 4(a)). At the 0.5 h time point, rasfonin-induced autophagy was completely inhibited in RIP1-depleted cells, as CQ failed to further accumulate LC3-II under the scenery (Figure 4(a)). Compared to mock-control, rasfonin induced less autophagic flux in RIP1-depleted cells at the 1 h time point (Figure 4(a)). Nevertheless, the induced autophagy was no longer inhibited at the 2 h time point (Figure 4(a)). The aforementioned results indicated that RIP1 mediated rasfonin-induced autophagy depending on stimulation duration. Similar to Nec-1 treatment, RIP1 deprivation enhanced rasfonin-induced PARP-1 cleavage (Figure 4(b)), indicating that the inhibition of RIP1 increased caspase-dependent apoptosis by rasfonin. Using MTS assay, we found that rasfonin induced more cell viability loss in RIP1-depleted cells (Figure 4(c)).

## Discussion

One of the processes by which normal cells become cancerous involves the deregulation of PCD (Sun and Peng 2009; Hanahan and Weinberg 2011). Given that there is no apparent cause and, that targeting only one pathway does not generally generate quantifiable improvement, cancerous growth is one of the most difficult diseases to treat (Gong et al. 2014). Therefore, multiple pathways must be targeted at the same time in order to have a truly effective cancer therapy (Gong et al. 2014). In recent years, more and more people have recognised that autophagy is vital for cancer development and treatment (Berardi et al. 2011). Thus, targeting autophagy in cancer will provide very promising opportunities for new drug development. Nevertheless, there are few studies on autophagy and fewer studies discussing autophagy and drug development in renal cancer. In a current study, we revealed that rasfonin activates mixed PCD, including autophagic cell death, apoptosis and necroptosis.

Usually, necrosis is believed to be unregulated; recently, a new kind of programmed necrosis has been discovered and invented as necroptosis (Cho 2014). In our former study (Zhang et al. 2011), we reported that 11ʹ-deoxyverticillin A (C42), a member of a class of fungal secondary metabolites known as epipolythiodioxopiperazines (ETPs), could induce autophagy and necrotic cell death of tumour cells through RIP1. Similarly, in this study, we found that the inhibition of necroptosis increased caspase-dependent apoptosis by rasfonin, and affected the induced autophagy depending on stimulation duration. Thus, it is likely that RIP1 mediates the switch between cell deaths related to stimulus type.

Although the exact reason for this phenomenon is unknown, we speculated that the inhibition of RIP1 might affect these signalling pathways that regulated the autophagic process. For we have observed that Nec-1 was able to increase AMP-activated protein kinase (AMPK) signalling, a main pathway that participated in autophagy regulation (Mihaylova and Shaw 2011), and the inhibition of AMPK either chemically or genetically affected rasfonin-induced autophagy similar to that of Nec-1 did (data not shown). Therefore, future works are needed to illuminate whether Nec-1 regulates autophagy through mediating AMPK pathway.

In summary, we revealed the rasfonin-induced mixed types of PCD, among which there existed an intricate cross-talk. Besides necroptosis, RIP1 was actively involved in rasfonin-induced autophagy as well as apoptosis. The data presented here and future work in this direction will provide one important clue for potential drug development from fungal secondary metabolites in renal cancer treatment.

## Materials and methods

### Chemicals and antibodies

Chloroquine diphosphate salt (CQ, C6628), necrostatin-1 (Nec-1, N9037), and polyclonal antibodies against LC3 (L7543) were purchased form Sigma-Aldrich (St. Louis, MO, U.S.A.). Antibodies against PARP (9542) and p44/42 MAPK (total-Erk1/2, 9102) were purchased from Cell Signaling Technology (Beverly, MA, U.S.A.). p62 (sc-28359) was acquired from Santa Cruz Biotechnology (Santa Cruz, CA, U.S.A.). Antibody against actin (TA-09) was obtained from Zhong Shan Jin Qiao Biocompany (Beijing, China). MTS reagent powder (G1111) was acquired from Promega Corporation (Madison, WI, USA).

### siRNAs

The siRNA specific for human RIP1 (SC-36426) was purchased from Santa Cruz Biotechnology along with the control siRNA (sc-37007).

### Cell culture and immunoblotting analysis

ACHN cell lines were grown in DMEM medium containing 10% foetal bovine serum (GIBCO), and 1% antibiotics. Cells were grown to 70% confluence before adding varieties of compounds. Whole cell lysates were prepared with lysis using Triton X-100/glycerol buffer, containing 50 mM Tris-HCl, pH 7.4, 4 mM EDTA, 2 mM EGTA and 1 mM dithiothreitol, supplemented with 1% Triton X-100, 1%SDS and protease inhibitors and then separated on an SDS-PAGE gel and transferred to PVDF membrane. Immunoblotting was performed using appropriate primary antibodies and horseradish peroxidase-conjugated suitable secondary antibodies, followed by detection with enhanced chemiluminescence (Pierce Chemical).

### Cell viability assay (MTS)

Cells were plated in 96-well plates (5000 cells per well) in 100 µl complete culture medium. After overnight culture, the medium was replaced with Phenol Redfree complete medium that was either drug-free or contained rasfonin or other chemicals. The cells were cultured for various periods and cellular viability was determined with CellTiter 96 Aqueous Non-Radioactive Cell Proliferation Assay (Promega).

### Immunofluorescence

ACHN cells were plated on glass coverslips and then the indicated treatment was performed. Then the cells were washed with Ca^2+^- and Mg^2+^-free PBS (CMF-PBS), fixed with freshly prepared 4% paraformaldehyde and permeabilised by incubation with CMF-PBS containing 0.1% TritoX-100 and 0.5% BSA. Then washed three times with CMF-PBS, and incubated with indicated antibodies in the presence of 0.1% TritoX-100 and 0.5% BSA. After they were washed for three times, the cells were incubated with the secondary antibodies (Alexa Fluor® 594 Goat anti-Rabbit and Alexa Fluor® 488 Goat anti-Mouse) diluting in CMF-PBS containing 0.5% BSA. The cells were then immersed in VECTASHIELD with DAPI (H1200, Burlingame, CA, USA) to visualise the nuclei after washing for three times. Images were acquired via fluorescence microscopy.

### Flow cytometry assay

ACHN cells were treated with the indicated compounds, then trypsinised and harvested, washed with PBS buffer, followed by incubating with a fluorescein isothiocyanate-labelled annexin V (FITC) and propidium iodide (PI) according to the instructions of an Annexin-V-FITC Apoptosis Detection Kit (Biovision Inc., K101-100) and analysed by flow cytometry (FACSAria, Becton Dickinson).

### Electron microscopy

Electron microscopy was performed as described. Briefly, samples were washed three times with PBS, trypsinised, and collected by centrifuging. The cell pellets were fixed with 4% paraformaldehyde overnight at 4°C, post-fixed with 1% OsO4 in cacodylate buffer for 1 h at room temperature, and dehydrated stepwise with ethanol. The dehydrated pellets were rinsed with propylene oxide for 30 min at RT and then embedded in Spurr resin for sectioning. Images of thin sections were observed under a transmission electron microscope (JEM1230, Japan).

### Statistical analysis

Several X-ray films were analysed to verify the linear range of the chemiluminescence signals and the quantifications were carried out using densitometry. Normally distributed data are shown as mean ± SD and were analysed using one-way analysis of variance and the Student–Newman–Keuls post hoc test. Data are shown as mean ± SD in the graphs.
