# Supplementary material for: Fungal secondary metabolites rasfonin induces autophagy, apoptosis and necroptosis in renal cancer cell line
Source: Mycology. 2016 May 9;7(2):81–7. doi: 10.1080/21501203.2016.1181114 (PMC6059062; doi:10.1080/21501203.2016.1181114)
Supplement: Supplementary_material.pdf [file TMYC_A_1181114_SM2285.pdf]

Supplement date

A

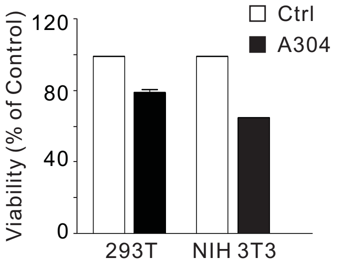

Figure legend: MTS was applied to detect the viability of 293T and NIH 3T3 cells after treatment with rasfonin (6μM) for 24 h. Data are presented as mean ± S.D. and are representatives of three independent experiments. Each performed in triplicate. It shows that the two cells viability underwent a losses of 20% and 60% respectively.
